# VEGF 936C > T Polymorphism and Association of BI-RADS Score in Women with Suspected Breast Cancer

**DOI:** 10.4137/bcbcr.s3164

**Published:** 2009-10-06

**Authors:** M. Wehrschuetz, H. Schöllnast, E. Wehrschuetz, W. Renner, G. Luschin

**Affiliations:** 1 Department of Radiology, Medical University Graz, Graz, Austria; 2 Institute of Medical and Chemical Laboratory Diagnostics, Medical University Graz, Graz, Austria; 3 Department of Obstetrics and Gynecology, Medical University Graz, Graz, Austria

**Keywords:** vascular endothelial growth factor, VEGF, breast cancer, polymorphism

## Abstract

**Purpose:**

Vascular endothelial growth factor (VEGF) is a potent regulator of angiogenesis and thereby involved in the development and progression of solid tumors. A 936C > T polymorphism in the VEGF gene has been associated with reduced VEGF plasma levels. Purpose of the present study was to analyze the potential association between VEGF genotype and radiological appearance of breast lesions by mammography.

**Materials and Methods:**

Fifty two women with 54 suspected breast lesions were analyzed by the use of mammography with the standard breast imaging reporting and data systems (BI-RADS). Germline VEGF genotype was determined in all subjects by allele-specific digestion of amplification products. An open biopsy was performed on all lesions.

**Results:**

VEGF CC, CT and TT genotypes were found in 41 (79%), 9 (17%) and 2 (4%) patients. By mammography 26, 16 and 12 suspected breast lesions were classified as BI-RADS scores 3, 4 and 5, respectively. Both carriers of the TT genotype were classified as BI-RADS 5, whereas among CT or CC carriers, BI-RADS scores 3, 4 and 5 were found in 26, 16 and 10 subjects (P < 0.026).

**Conclusion:**

The VEGF 936C > T polymorphism seems to be associated with a high BI-RADS score in women with suspicious breast lesions.

## Introduction

Vascular endothelial growth factor (VEGF) is an important regulator of vasculogenesis and angiogenesis with a specific mitogenicity for endothelial cells.^1^,^2^ In addition, VEGF can increase capillary permeability, dilate arteries and attract monocytes chemotactically.^3^,^4^ VEGF is a disulfide-bonded dimeric glycoprotein, sharing close sequence homology with VEGF-B and VEGF-C and placenta growth factor, and lower sequence homology with platelet-derived growth factor.^5^,^6^ Strong expression of VEGF has been observed in a variety of tissues, including tissues of the female reproductive system, ischemic tissues, tumours and transformed cell lines.^7^

Breast cancer is the most frequently diagnosed cancer in Western societies, with a lifetime incidence of about 10%–13% among women.^8^,^9^ Mammography is the accepted method for screening to diagnose small breast cancers. The present study intended to analyze the potential association between VEGF genotype and the radiological/mammographic assessment of breast lesions.

## Materials and Methods

### Subjects

Fifty-two women (age range, 30–82; mean age, 54.5 +/− 11.8 years) were prospectively enrolled in our trial between October 2002 and March 2003. Inclusion criteria were 1) breast symptoms, 2) no history of breast cancer, 3) female sex, 4) written informed consent. Exclusion criteria were 1) history of breast cancer, 2) pregnancy and 3) age under 18 years. All women had a full diagnostic work up including clinical examination, mammography, breast ultrasound and all had a preoperative localisation procedure under stereotactic or ultrasound guidance. Each woman provided a blood sample preoperatively for further genetic analysis. Each sample was marked with a serial number to assure data protection of personal data. The study was approved by the ethics committee of the Medical University of Graz. TNM classification, lymph node status, ER, PR and Her2/neu were routinely defined by our institute of pathology and elaborated for the included patients by the study authors.

## Statistical Analysis

P-values for the comparison of the BI-RADS III, IV, V and VEGF 936C > T polymorphism and the comparison of VEGF 936C > T polymorphism and TNM classification, lymph node status, ER, PR and Her2/neu were calculated with Pearson’s Chi square test and Wilcoxon-Mann-Whitney test using the exact test option in StatXact 4.0.1 (Cytel Software Corp., Cambridge, MA). Threshold for significance was p < 0.05.

## Imaging and Evaluation

Mammography was performed with a Siemens Mammomat 3000 (Siemens Medical System, Erlangen, Germany). A dedicated Agfa film processor with an extended (2-minute) processing cycle was employed with Adefo chemistry at 34 °C development temperature. A commercial single-emulsion mammography film with standard screens and carbon cassettes were used (previously MicrovisionCi, Sterling, USA—now Agfa, Belgium) at a mammography site approved by the Austrian Ministry of Public Health. Two experienced radiologists analyzed the mammography images. All images were classified by the standard breast imaging reporting and data systems (BI-RADS).^10^ Breast ultrasound was performed at the discretion of the radiologists with a Logiq 700 MR (General Electric Medical System, Milwaukee, USA). A digital system (OPDIMA, Siemens Medical System, Erlangen, Germany) was used for preoperative stereotactic localisation.

A microbiologist specialised in genetics determined the patients’ genotypes as follow:

For genetic analyses, genomic DNA was isolated from venous blood by standard methods and stored at 4 °C. A 198 bp fragment containing the polymorphic site was amplified by polymerase chain reaction (PCR) using 5′-AAGGAAGAGGAGACTCTGCGC-3′ as forward and 3′-TATGTGGGTGGGTGTGTCTAC AGG-3′ as reverse primer. The PCR product was digested with restriction endonuclease NIaIII (New England Biolab); fragments were analyzed on 2.0% agarose gels stained with ethidium bromide. The C allele remained uncut, while the T allele was cut into two fragments of 114 and 84 bp. For each set of reactions, a negative control containing H_2_O instead of DNA to check for contamination and a positive control (sample with homozygous TT genotype) to check for complete digestion were added.

## Results

**Figure 1.**
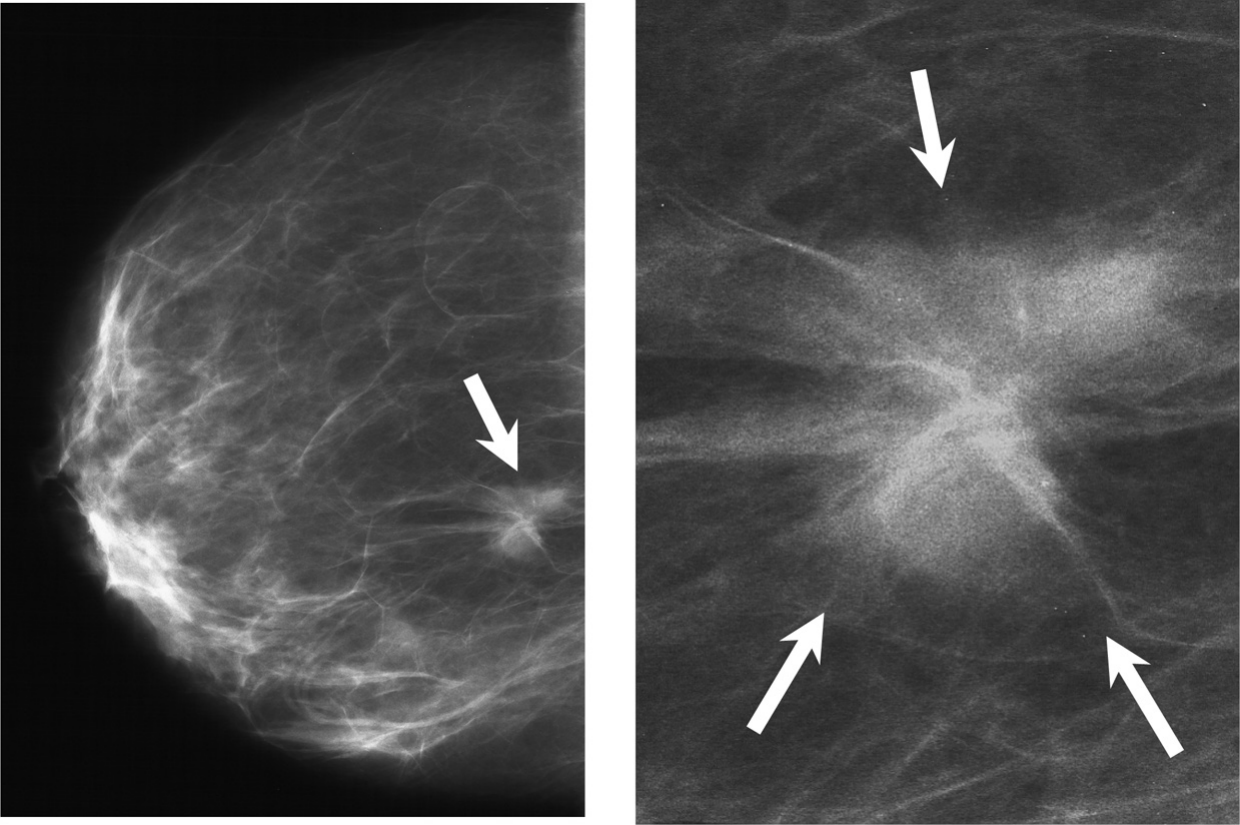
Obscure mass due to infiltrating ductal carcinoma (pT1cN0G3) and branching and/or fine linear calcifications (arrow) due to infiltrating ductal carcinoma and ductal carcinoma in situ in the mediolateral view in a 47-year-old woman. BI-RADS classification V; The patient carries the TT allel.

**Figure 2.**
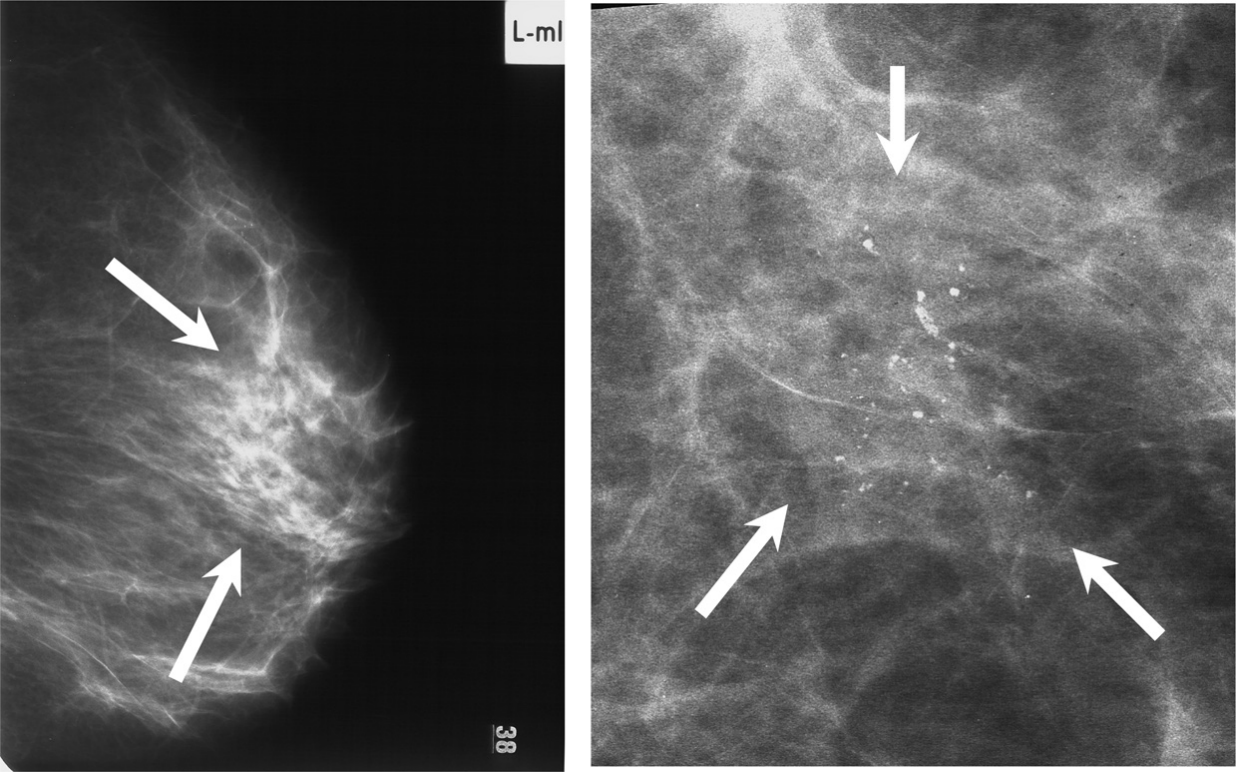
Branching and/or pleomorphic calcifications (arrow) in mediolateral view due to ductal carcinoma in situ (pTisG2) in a 51-year-old woman. BI-RADS classification IV. The patient carries the CC allele.

The study consisted of 52 women with 54 breast lesions. VEGF genotype was determined for all subjects. With mammography, 26, 16 and 12 suspected breast lesions were classified as BI-RADS category 3, 4 and 5, respectively. VEGF CC, CT and TT genotypes were found in 41 (79%), 9 (17%) and 2 (4%) patients. The two carriers of the TT genotype were classified as BI-RADS 5 Figures 1 and 2; among CT or CC carriers, BI-RADS scores 3, 4 and 5 were found in 26, 16 and 10 subjects, respectively (p < 0.026) (Table 1). In all, 28 lesions (51.8%) were considered to be cancerous. No correlation was found between the 936C > T gene polymorphism of VEGF and tumor size and histological grading (p < 0.83), lymph node status (p < 0.54), estrogen receptor status (p < 1.1), progesterone receptor status (p < 0.76), or HER2/neu receptor status (p < 0.8) (Tables 2, 3). The sensitivity and the specificity were 86% and 97%, respectively, with 92% accuracy. Histological diagnosis was the gold standard.

## Discussion

The aetiology of breast cancer is still not fully understood. Besides age at menarche and menopause, diet, reproductive history, oestrogen administration and genetic factors have been suggested as risk factors.^11^–^15^ Many women will at some time have breast symptoms, especially between 40–70 years of age; the risk of developing breast cancer is gradually increasing in this age range. During a given 10-year period, it is estimated that 13%–16% of women will seek medical advice due to breast symptoms.^16^,^17^

Our intention was to analyze the potential association between VEGF genotype and mammography findings to provide information to supplement the clinician’s repertoire of breast palpation, imaging techniques, and biopsy.

Appropriate management of breast findings is very important for the primary physician regarding a reported diagnosing rate for breast cancer of 4% of patients with breast symptoms.^16^ Pooled data from screening studies estimate sensitivity of 54%.^18^ There is considerable interobserver variability in detection rates and interpretation. Only 25% interobserver agreement was shown in a study involving experienced breast surgeons who evaluated breast abnormalities in 100 consecutive women with breast symptoms. Biopsy was recommended in 28%–39% of these cases, but only in 17% were the surgeons in agreement.^19^

With breast induration, Kaiser et al found that mammography alone had a sensitivity of 60% for invasive breast carcinoma. The specificity of mammograms upon initial imaging was 94% and the negative predictive value was 97%.^20^ The usefulness of breast US in detecting carcinomas was demonstrated by Butler et al,^21^ who detected 87.7% of mammographically subtle or invisible lesions.

We find that it can be very difficult to evaluate breast symptoms by palpation, especially in patients who have previously undergone biopsy procedures; these can entail postsurgical sequelae such as breast scarring, fat necrosis, architectural distortion and skin changes that can mislead the radiologist. We so hypothesized that VEGF in general and the 936C/T polymorphism in particular could help radiologists resolve these ambiguities.

In our cohort, both carriers of the TT genotype were classified as BI-RADS 5, whereas among CT or CC carriers, BI-RADS scores 3, 4 and 5 were found in 26, 16 and 10 subjects, respectively (P < 0.026). Carriers of the TT allele with breast symptoms thus face a significantly higher risk for breast cancer than those without that allele and, if supported by mammography and ultrasound, should be strongly urged to undergo biopsy due to their greater likelihood of having breast cancer with a high-grade BIRADS than patients carrying the CC or the CT allele, as suggested by the literature.^22^ Erolglu et al failed, however to find an association between the 936C/T polymorphism and clinicopathological characteristics,^23^ although it has been demonstrated beyond reasonable doubt that VEGF plays an important role in breast cancer,^24^ and there appears to be a significant positive association between HER-2/neu and VEGF expression.^25^ In any case, as our data show statistical significance in the comparison of 936C/T polymorphism and BIRADS scores in mammography, we propose a potential association of the 936C/T polymorphism with mammographic appearance of breast lesions in women suffering breast symptoms.

A limitation of our study is its small size. Further prospective trials should clarify the role of VEGF and potential morphologic coherences of the VEGF 936C > T polymorphism and the mammographic appearance in women with breast symptoms, also taking into account diet, hormonal status and other life-style factors that are known to contribute to mammographic density.

## Figures and Tables

**Table 1.** Comparison of BI-RADS III, IV, V and VEG F 936C > T polymorphism in patients with breast symptoms.

	**BI-RADs III**	**BI-RADs IV**	**BI-RADs V**	
Allele				
CC + CT	26	16	10	
TT	0	0	2	p < 0.026

p < 0.05.

**Table 2.** Comparison of VEGF 936C > T polymorphism and TNM classification.

	**TT (%)**	**CC + CT (%)**
pTIS, n (%)	2 (7.1)	6 (21.4)
pT1, n (%)	0 (0)	17 (60.7)
pT2, n (%)	0 (0)	1 (3.5)
pT3, n (%)	0 (0)	2 (7.1)
pT4, n (%)	0 (0)	0 (0)
Nx, n (%)	1 (3.5)	15 (53.5)
N0, n (%)	1 (3.5)	8 (28.5)
N1, n (%)	0 (0)	3 (10.7)
N2, n (%)	0 (0)	0 (0)
N3, n (%)	0 (0)	0 (0)
N4, n (%)	0 (0)	0 (0)

p < 0.83; p < 0.54.

**Table 3.** Comparison of VEGF 936C > T polymorphism and receptor status.

	**TT (%)**	**CC + CT (%)**
Estrogen receptor pos., n (%)	2 (7.1)	22 (78.5)
Estrogen receptor neg., n (%)	0 (0)	4 (14.2)
Progesteron receptor pos., n (%)	2 (7.1)	14 (50)
Progesteron receptor neg., n (%)	0 (0)	12 (42.8)
HER2NEU pos., n (%)	0 (0)	6 (21.4)
NER2NEU neg., n (%)	2 (7.1)	20 (71.4)

p < 11; p < 0.8; p < 0.76.
